# Constructing the Corpus of Children’s Video Media (CCVM): A new resource and guidelines for constructing comparable and reusable corpora

**DOI:** 10.3758/s13428-025-02925-7

**Published:** 2026-01-23

**Authors:** Anna Gowenlock, Jennifer Rodd, Beth Malory, Courtenay Norbury

**Affiliations:** 1 Division of Psychology and Language Sciences, UCL, London, UK; 2 Department of English Language and Literature, UCL, London, UK; 3 Department of Clinical, Educational, and Health Psychology, UCL, 26 Bedford Way, London, WC1H 0AP UK; 4 Department of Experimental Psychology, UCL, London, UK

**Keywords:** Video media, Vocabulary, Language development, Corpus linguistics

## Abstract

A growing number of psycholinguistic studies use methods from corpus linguistics to examine the language that children encounter in their environment, to understand how they might acquire different aspects of linguistic knowledge. Many of these studies focus on child-directed speech or children’s literature, while there is a paucity of work focusing on children’s television and video media. We describe the creation and contents of the Corpus of Children’s Video Media (CCVM), a specialised corpus designed to represent the spoken language in television and online videos popular among 3–5-year-old children in the UK (available as a scrambled database of tokens). The CCVM was designed to be comparable to an existing corpus of child-directed speech (CDS). We used a dual sampling approach: inclusion decisions were guided by (a) a survey of parents with children in our target age group, and (b) a survey of programmes available on popular streaming platforms. The corpus consists of 233,471 tokens across 161 transcripts (43.12 h of video) and is available on the Open Science Framework (OSF) as a scrambled database of tokens (including gloss, stem, and lemma forms, and part-of-speech tags), organised within transcripts, together with relevant metadata for each transcript. We discuss the challenges of creating a corpus that is comparable to existing datasets and highlight the importance of transparency in this process. We take an open science approach, sharing a detailed data collection and processing protocol, code, and data so that the corpus can be evaluated, extended, and used appropriately by other research teams.

## Introduction

Children learn about words and their meanings by encountering them in context, including through child-directed speech (Anderson et al., 2021; Rowe, 2012), shared book reading (Farrant & Zubrick, 2012; Dowdall et al., 2020; Biemiller & Boote, 2006), and video media (Kaefer et al., 2025; Neuman et al., 2020; Rice & Woodsmall, 1988). Corpus studies allow researchers to examine and compare the types and complexity of language that children are exposed to in these different contexts, to better understand the vocabulary learning opportunities afforded by different types of input. For example, existing studies show that children’s picture books contain vocabulary that is more diverse, sophisticated, and structurally complex than the vocabulary of child-directed speech (Montag et al., 2015; Montag, 2019; Dawson et al., 2021, Nation et al., 2022); therefore, experience with print might be an important, and perhaps unique, source of lexical knowledge even for very young children. This is an important topic given that early vocabulary is predictive of important later skills including reading and academic attainment (Nation & Snowling, 2004). Unlike book language, the language of children’s video media remains poorly understood, despite the fact that children typically spend multiple hours a day watching videos (Ofcom, 2024; Rideout & Robb, 2020). Here we present the Corpus of Children’s Video Media (CCVM), a newly developed, specialised resource constructed to characterise the linguistic opportunities provided by video media.

The guiding principle when constructing this corpus was that it should facilitate comparisons with other sources of linguistic input, such as day-to-day conversations with caregivers. Specifically, we chose to make this corpus closely comparable to the child-directed speech (CDS) data available through CHILDES (MacWhinney, 2000), in terms of both the sampled age range and the details of transcript processing and formatting. The utility of such between-corpus comparisons is demonstrated by Dawson et al. (2021), who constructed a specialised corpus of picture books aimed at children aged 0–7 years and compared this to a corpus of CDS drawn from CHILDES. They analysed three metrics of lexical richness: lexical sophistication (the proportion of common words in each corpus), lexical density (the proportion of meaning-rich words to function words in each text), and lexical diversity (the proportion of unique words to total words in each corpus). By comparing these three metrics across books and conversation, Dawson and colleagues concluded that picture books contain richer vocabulary than day-to-day conversations with caregivers. In this project, we build a novel corpus of children’s media, aiming to provide a key resource that will allow for similar insights into the characteristics of the video media that is typically encountered by young children in the UK and how video media compares to other sources of child language input. Specifically, this corpus was developed for a project that extends the lexical richness analyses of Dawson et al. (2021), including comparisons of lexical diversity, density, and sophistication across video media and CDS. This paper does not present the results of this analysis, instead focusing on the methodological issues that arise when building a corpus that is comparable to an existing dataset.

### Corpus matching considerations

When making comparisons between corpora, the two corpora should ideally differ only in the factor of interest (in this case, medium), with all other factors controlled and held constant. As Ädel (2020) writes: “In order for the comparison to be valid … the two sets ((sub-)corpus A and (sub-)corpus B) need to be maximally comparable with regard to all or most factors, except for the one being contrasted” (p. 17). This careful matching between corpora would likely be best achieved by a single research team constructing both corpora under the same conditions. However, given the significant resource requirements of compiling a corpus, many corpus comparison studies choose to make use of pre-existing data, yielding corpora that are constructed by different people, at different times, and for different purposes. While this increases the efficiency and feasibility of corpus research, it makes the task of careful matching between corpora more difficult, particularly if pre-existing corpora lack clear documentation. Matching needs to occur at all stages of corpus design, from defining the target domain that the corpus aims to represent, to the choice of software used to automatically process and tag corpus data. Below we provide examples of how poor matching might arise at different stages of the corpus compilation process and the potential influence on study conclusions.

#### Stage: Defining the operational domain of the corpus

The operational domain of a corpus is the set of available texts from which the final set of texts is sampled (Egbert et al., 2022). Differences in the way that variables are defined in the operational domain can introduce poor matching between corpora. One clear example from developmental research is the difficulty in assigning “target age” to different media and applying age cut-offs to the operational domain. For CDS transcripts, target age is relatively straightforward: we can simply take the age of the child that is present in the recording as the target age of each transcript. It is more complicated to define target age for child-directed media, since a single book or TV show might be designed for children of a range of ages and may be consumed by an even broader age group. Since age is necessarily defined differently for CDS than for books or videos, ensuring that the corpora are well matched on age is challenging. For example, the Picture Book corpus created by Dawson et al. (2021) is composed of texts that were aimed at 0–7-year-old children according to retailer bestseller lists and recommendations from charities, websites, and teachers. However, it is possible to define the target age of each book in other ways (e.g. through the advertised target age group for each book or by surveying parents). The way “target age” is defined therefore plays an important role in determining the operational domain and may result in poor age matching between corpora. Additional complexities arise when trying to determine whether different corpora are comparable in terms of socioeconomic status (SES, e.g. Gathercole, 1986). Although these issues cannot be easily resolved, they highlight the importance of clearly reporting how key text characteristics are defined and how the limits of the operational domain are determined.

#### Stage: Data collection

During data collection, differences in the tools and systems used can also result in poor matching between corpora. Particularly in the case of manual speech transcription, it is important to minimise systematic differences in transcription style that could impact analysis. For example, arbitrary differences in spelling between corpora (e.g. the choice between American and British spellings) may give the false impression that certain words appear more frequently in one corpus than in the other. In cases where transcription relies on more subtle judgements (e.g. distinguishing between interjections such as “ah” and “aah”), corpora may not be internally consistent, making the task of matching between corpora even more difficult (Andersen, 2016). Transparent guidelines are also needed to ensure consistency in the coding of, for example, abbreviations (e.g. “goin” vs “going”), acronyms (e.g. “U_S_A” vs USA”), letter names, foreign words, neologisms, singing, and onomatopoeias (see the Data Collection Protocol on the OSF, 10.17605/OSF.IO/GRBYX). Transparency about the tools and systems used to construct the original corpus is therefore crucial both for selecting consistent systems for the new corpus (e.g. British or American spellings; systems for abbreviations) and for understanding which elements of the data may be unreliable (e.g. interjections).

#### Stage: Data processing

Once texts have been identified and transcribed, there are several data processing steps that may be completed automatically (e.g. assigning part-of-speech tags) or manually (e.g. categorising or annotating examples for analysis). In both cases, it would be optimal for the same procedures and tools to be used for both corpora, since different tools are unlikely to produce identical results (Newman & Cox, 2020). For example, Newman and Cox (2020) examine the treatment of the word “rid” across four different part-of-speech taggers and find that all four taggers produce different results. Differences in data processing may therefore produce spurious results that reflect arbitrary differences between the corpora, rather than differences relating to the factor of interest.

The examples above highlight that clear reporting is necessary for assessing how well two corpora are matched on key design features (e.g. the age range and SES associated with the texts), data collection procedures (e.g. transcription style), and processing steps (e.g. part-of-speech tagging). Clear reporting is also necessary to allow other researchers to evaluate how well a corpus represents its target domain (Egbert et al., 2022). Without this clarity, researchers may compare corpora without fully understanding their biases or limitations and how these might influence the study results.

In this paper, we therefore describe in detail the construction and contents of the Corpus of Children’s Video Media. We transparently document the corpus compilation process for two key reasons:

1. To demonstrate that our corpus is comparable to the CDS data, and
2. To facilitate the evaluation and re-use of our corpus by other researchers.

We adopt additional open science principles for corpus studies (Hartmann, 2024; Sönning & Werner, 2021) by preregistering our study and sharing extensive online materials that provide a practical example of the creation of a specialised corpus for researchers new to corpus linguistics and those looking to make cross-corpora comparisons.

The paper is structured as follows: First we outline the key requirements for the CCVM. These are, in part, based on the expectation that matching to existing CDS corpora (MacWhinney, 2000) is key to its future utility. We review existing corpora of children’s video media to confirm that no existing corpus meets these requirements, before describing the corpus construction. In the methods section, we describe our sampling and data collection procedures, along with additional processing steps applied to both the CCVM and the CDS corpus. We then describe the contents of the CCVM before finally discussing the limits and appropriate uses of this corpus and the extent to which the CCVM is comparable to the CDS corpus.

### Video corpus requirements

The general requirements for the Corpus of Children’s Video Media are as follows:

- The corpus should be representative of programmes watched by *children aged 3–5 in the UK*. The lower bound to our age range was selected because of evidence that children below the age of 3 years do not learn vocabulary effectively from video media (Roseberry et al., 2009; Jing et al., 2023). The upper age bound was chosen because the available CDS transcripts rarely include children over the age of 5 (MacWhinney, 2000).
- The programmes should be *popular and readily available* for children in our age range. Note that this does not necessarily mean that all programmes must be child-directed, since it is possible that children are watching programmes that are not explicitly created for their age group.
- The corpus should be structured as a *collection of transcripts*, rather than a database of lexical statistics. This allows for analysis at the level of individual transcripts rather than only making generalisations across the corpus as a whole. It also allows us to examine the link between lexical richness and properties of specific episodes (e.g. narrative style).

In addition to these general requirements, the corpus should be matched to the subset of a broader CDS database that we identified as being suitable for future between-corpus comparisons. Transcript data are taken from the Eng-UK section of CHILDES, a large online database of child speech and child-directed speech (MacWhinney, 2000, https://childes.talkbank.org/access/Eng-UK/). The Eng-UK section contains data from 22 studies covering a range of settings and participants across the UK, from which we selected 734 suitable transcripts across eight studies^1^. Each transcript was available to download from CHILDES in .cha format, already tagged with parts of speech. The resulting corpus contains a total of 2,588,897 tokens. The data were collected in a broad range of locations (including Northern Ireland, Scotland, and England) between 1973 and 2008. Recordings were typically made during unstructured play sessions and included speech from caregivers, relatives, researchers, and other children. In particular, we ensured that the CCVM was compatible with the CDS data in the following ways:

- The same age range is used for both corpora (3;00–5;11).
- The corpus is transcribed according to the same guidelines as the CDS data (CHAT format, MacWhinney, 2000, https://talkbank.org/).
- The corpus is processed and tagged using the same software used to process the CDS transcripts (CLAN, MacWhinney, 2000, https://talkbank.org/).

### Existing corpora of children’s video media

After defining these requirements, we first checked to see whether there were any suitable pre-existing corpora that were publicly available. We found that, while there have been recent studies examining children’s video media (Neuman et al., 2019; Brodsky & Sulkin, 2021; Green, 2021), existing corpora were either not publicly available or did not meet the requirements above. To the best of our knowledge, there are two large publicly available corpora that contain subtitles from children’s TV and films. These are the TV Corpus (Davies, 2021) and the SubIMDB corpus (Paetzold & Specia, 2016). Both corpora contain tens of thousands of subtitles extracted from OpenSubtitles (https://www.opensubtitles.com/en), all linked to metadata from IMDb (https://www.imdb.com/). A subsection of the SubIMDB corpus has previously been used to examine the vocabulary learning opportunities in children’s TV and film (Green, 2021). While both of these corpora are vast, neither met our requirement that the corpus be representative of programmes watched by children aged 3–5 in the UK. Although the corpora could be filtered by genre or age rating, in practice these filters are not sufficiently restrictive to ensure the corpus represents the language domain we are interested in. For example, the TV Corpus can be filtered to only show programmes that have a rating of TV-Y, meaning that they are appropriate for all children. However, this category is likely to contain programmes that are not popular among our target age group (3–5 years). It may also exclude programmes that are popular among this group despite being inappropriate due to higher age ratings.

Another potential data source was the SUBTLEX-UK database (van Heuven et al., 2014). This is a database of lexical statistics derived from subtitles of 45,099 different broadcasts. SUBTLEX-UK contains word frequencies specifically for two child-directed TV channels (CBeebies and CBBC, aimed at children aged 0–6 and 6–12 years, respectively). The CBeebies data are a closer fit to our target domain in that the programmes are UK-based and specifically aimed at young children. However, data from a single broadcast TV channel are unlikely to be representative of the diverse viewing experiences of children today. Data are also organised as a lexical database rather than a corpus, meaning that we would not be able to conduct any analyses at the level of individual programmes. For example, comparisons such as the keyword analysis of Dawson et al. (2021) require word frequency counts that have been adjusted for the dispersion of the words within the corpus. Without access to raw transcript data, it would not be possible to investigate whether instances of individual words are clustered together in a small number of texts or distributed more evenly across the corpus. As none of the available corpora allow for these important between-corpus comparisons, we built a specialised corpus designed to be representative of our target domain and structured to facilitate token-level lexical analyses.

### Approach to corpus construction

To limit bias in the corpus building process, prior to data collection we preregistered key decisions about programme sampling, data collection, and processing, including the inclusion criteria for the videos. Preregistration is a useful tool to constrain the researcher degrees of freedom involved in corpus construction and analysis (Mak, 2024; Roettger, 2021).

Videos in the current corpus had to meet the following preregistered inclusion criteria:

- Available to watch in the UK on either live TV or online streaming services
- Professionally produced (rather than home videos or vlogs)
- Primarily in English (programmes dubbed in English were included)

A dual sampling approach was taken in which titles were selected based on (a) parent data about the programmes their children were viewing and (b) a survey of titles that were available/promoted to children on popular viewing platforms. The titles and episodes that were identified during these two surveys constituted the operational domain of the corpus (i.e. the set of programmes/videos from which the final sample was drawn). The parent survey may be biased towards those programmes that are more salient or that parents perceive as being more desirable. The platform survey captures those programmes that are available to children but not necessarily reported by parents. This dual approach therefore identifies programmes known to be *popular* within a sample of 3–5-year-olds (parent survey) and those most *readily available* to this age group (platform survey).

**Fig. 1:**
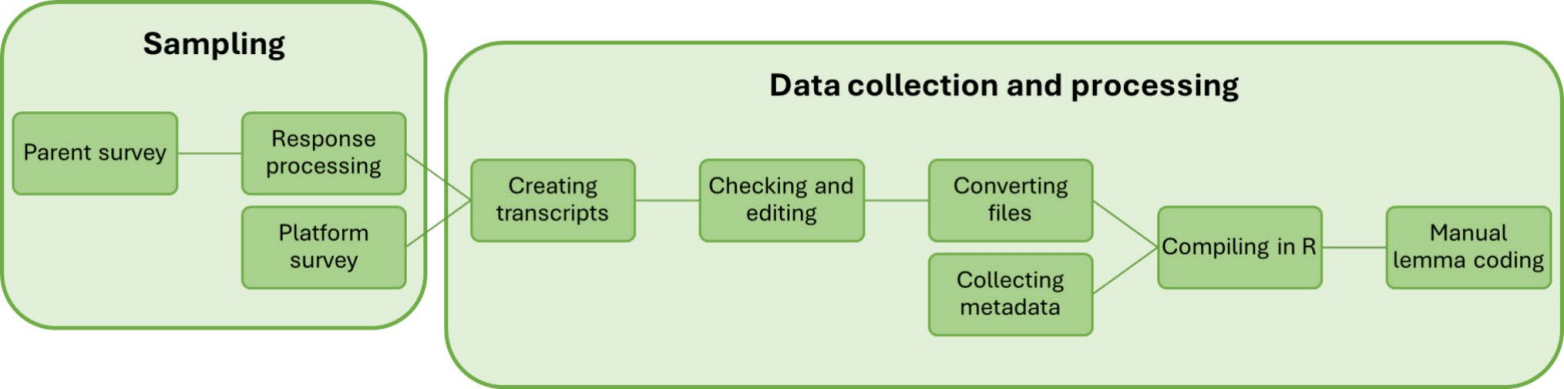
Data collection and processing pipeline

The full corpus construction pipeline is outlined in Fig. 1. Data processing steps follow Dawson et al. (2021). This allows the use of the same tagging software used in the comparison CDS corpus and for the data to be read into R (R Core Team, 2023). Once the video transcripts had been produced to match the format of the CDS transcripts downloaded from CHILDES (MacWhinney, 2000), all additional processing steps (file conversion, compilation in R, and manual lemma coding) were applied to the pooled data to ensure consistency across corpora.

## Corpus construction method

### Data availability

Scrambled token-level data from the CCVM are available on the OSF (*CCVM_Scrambled-tokens.csv*, 10.17605/OSF.IO/GRBYX). This database contains information about each token in the corpus including gloss, stem, and lemma forms, and part-of-speech tags. Tokens have been scrambled within each transcript for copyright reasons, meaning that the available data are suitable for count-based analyses (e.g. lexical frequency) but not sequence-based analyses (e.g. analysis syntax or turn-taking). Additional CCVM data shared on the OSF include a table of bigram frequencies (*CCVM_Bigram-frequency.csv*), a table of utterance length information within each transcript (*CCVM_Utterance-length.csv*), and metadata for each transcript (*CCVM_Transcript-metadata.csv*), including information about data collection (e.g. date of transcript creation, original source of the transcript), and features of the programme (e.g. series and episode number, programme and episode title, year of original release). We have also shared a step-by-step protocol for data collection, anonymised parent survey responses, R scripts to reproduce the figures and analysis in this paper, and a visual summary of the corpus.

### Programme sampling

#### Parent survey

##### Participants

Participants in the survey were 123 UK parents of 3-, 4-, and 5-year-olds recruited online through Prolific (https://www.prolific.com/) between 20/03/24 and 21/03/24. Of the 123 participants who completed the study, nine were rejected because they gave inconsistent information about their child’s age, leaving 114 participants in total (38 per age group). There were slightly more parents of male children in the sample (54.39%), particularly in the oldest age group (60.53%). This sample was more highly educated than the general population (Table 1), with 65.79% of the sample reporting degree-level education or above compared to 42.1% of UK parents in 2022 (Social Mobility Commission, 2024).

**Table 1:** Educational background of parents in our sample

Education	Count	Percent
Secondary school (up to 16 years)	8	7.02%
Further education (A-levels, BTEC, etc.)	31	27.19%
Undergraduate degree	52	45.61%
Postgraduate degree	23	20.18%

##### Methods

Participants were redirected from Prolific to Gorilla Experiment Builder (www.gorilla.sc), which hosted the survey (Anwyl-Irvine, et al., 2020). Participants provided informed, written consent online prior to questionnaire completion. Background information collected included child age, gender, and parent level of education. Parents were then asked to select which platforms their child had used to view videos in the last 2 weeks from a list of 10 options, and to report the titles of any programme their child watched during that period in a free text box. They were also asked to name the programme their child watched most often and indicate how often their child watched videos alone (5-point Likert scale ranging from *1 (never)* to *5 (every day)*). Participants typically completed the survey in 2–3 min.

Responses were coded according to category (e.g. film, comment, genre, programme, YouTube series) and the best matched programme. Best matches were selected by searching IMDb for the most popular and readily available child-directed programme match (e.g. “spiderman” would be matched to the children’s cartoon rather than the general audience live-action film). Where the parent’s response could be matched to multiple programmes (e.g. “Postman Pat” could match the original series from 1981 or the more recent *Special Delivery* adaptation), priority was given to recent, popular, and readily available programmes (i.e. the version that appeared on Netflix or YouTube, or the most recent iteration of a programme). Priority was also given to TV series over films where both were a viable match. Parent responses were then combined with child age in years and randomised within each age group. This randomly ordered list was used as the basis for sampling programmes to include in the corpus as described below.

##### Results

**Fig. 2:**
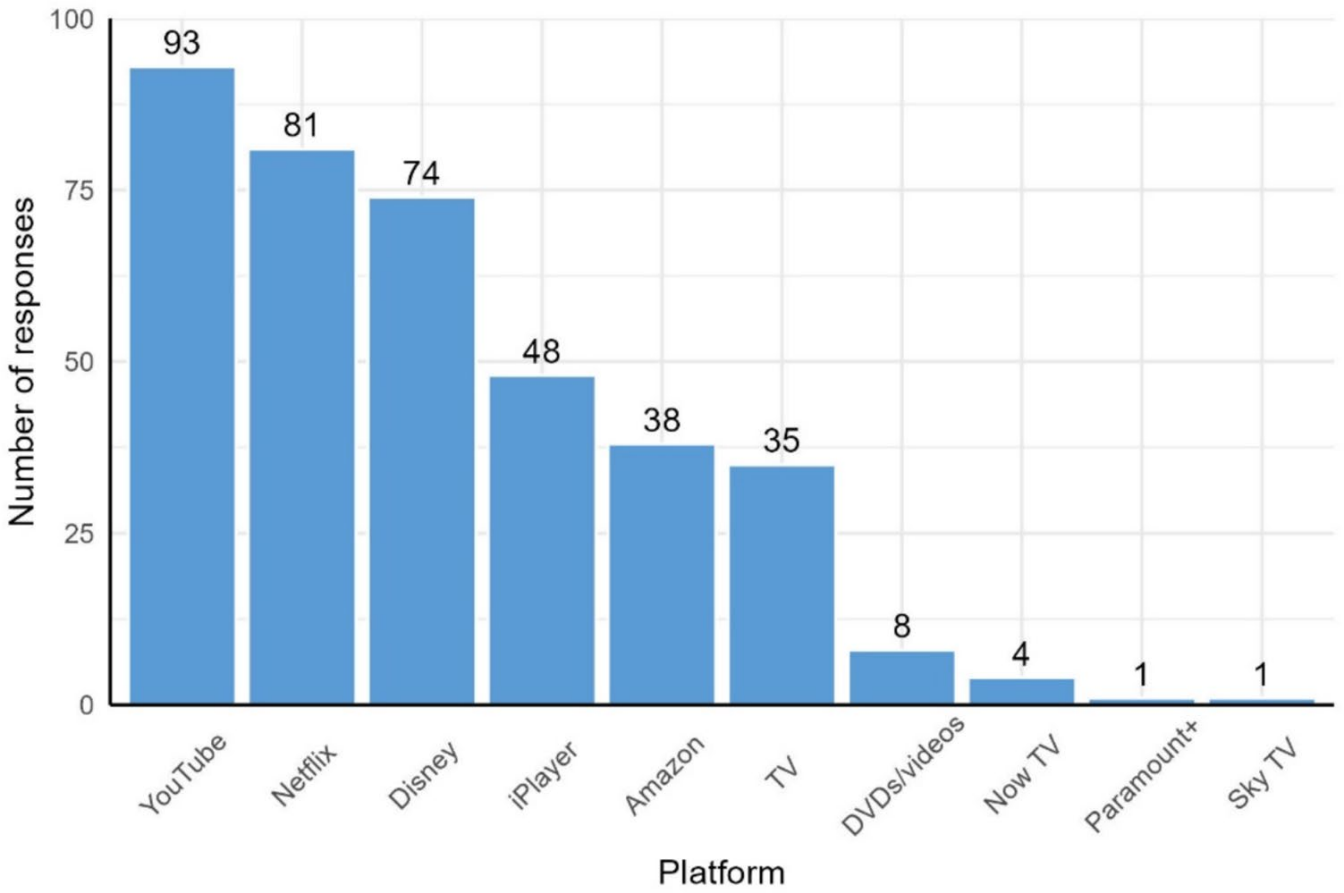
Number of parents who indicated that their child watched videos using each platform

Parents indicated that their children had watched an average of 3.36 different platforms (*SD* = 1.23) over the last 2 weeks (Fig. 2). The most popular platform was YouTube (watched by 81.58% of the sample), followed by Netflix (71.05%) and Disney+ (64.91%). The top five most popular platforms were used as the basis for the platform survey described below.

Parents provided 652 distinct responses to the question asking what programmes their child had watched recently. We removed responses that clearly did not meet the inclusion criteria according to metadata available online. A total of 104 responses (15.95% of all responses) were removed at this stage because they were in an irrelevant category (i.e. not a TV show or short video, *n* = 72), were not professionally produced (*n* = 3), could not be identified (*n* = 11), or were vlogs (*n* = 18)^2^. This left 548 responses: 499 (91.06%) referred to TV series, 38 (6.93%) referred to YouTube series or channels, and 11 (2.01%) referred to TV movies (i.e. short standalone films). *Bluey* was the most popular programme in all age groups, followed by *Peppa Pig* then *PAW Patrol* for 3- and 5-year-olds, and *PAW Patrol* then *Peppa Pig* for 4-year-olds.

##### Sampling method from parent survey

The output of the parent survey was a randomly ordered list of programme titles within each age group. We sampled programmes consecutively from these lists and completed final checks that they were eligible for inclusion in the corpus. Seven further responses were excluded at this stage on the basis that they did not meet the preregistered requirement to be “primarily in English” either because they contained no language (*N* = 6) or because they were in a language other than English (*N* = 1). Note that multiple episodes of a single programme could be included if the title appeared multiple times in the parent response list. This meant that more popular programmes (according to parents) contributed multiple episodes to the corpus.

Where a programme/channel did meet the criteria for inclusion in the corpus, the exact episode was sampled from one of four data sources in the following priority order:

1. A subtitle file downloaded from Open Subtitles (https://www.opensubtitles.com/en)
2. A transcript from Box of Broadcasts (BoB, https://learningonscreen.ac.uk/ondemand/)
3. A transcript from a YouTube video (https://www.youtube.com/)
4. A manual transcription of an episode available on a streaming platform

Beginning with the first source from the above list, we checked to see whether there were any episodes available from the specific programme to be included. Where episodes were available, we used a random number generator to randomly select a specific episode from the available set. If no episodes were available, we moved to the next data source from the list above. In effect, this meant that we were randomly sampling not from the entire body of existing episodes, but from the subset of episodes available from one of the above data sources. This method allowed us to prioritise episodes that had the most accurate transcriptions available online and minimise the amount of manual transcription. Our sampling method deviated from our preregistration in that when there was a very large number of unstructured results (e.g. when searching for a programme on BoB), an episode was selected from the first 10 pages of results only.

#### Platform survey

A platform survey was also conducted, as the parent survey indicated that 90.35% of the children in the sample watched media alone at least some of the time. The five most popular platforms from the parent survey were included (Netflix, YouTube, Disney+, BBC iPlayer, and Amazon Prime). The number of videos sampled from each platform was proportionate to the number of votes that platform received in the parent survey. For example, 93 out of the 334 votes (27.84%) for the top five platforms went to YouTube, so roughly 28% of the transcripts were collected from YouTube.

We aimed to sample only programmes that children were likely to have access to, even when browsing alone. We therefore created profiles on the five platforms with parental controls set up for a 4-year-old child (the middle of our age range). On Netflix, Disney+, YouTube, and Amazon Prime, we created a kids’ account and applied the suggested parental controls for a 4-year-old child. For BBC iPlayer, a normal adult account was used, but programmes were only sampled from the CBeebies channel page (a channel aimed at children aged 6 years and below). These parental restrictions are reasonably conservative, and some children may be watching alone on adult profiles with access to content not designed for their age group. While this may bias our sample towards well-designed child-directed programmes, sampling from unrestricted profiles would likely result in the inclusion of programmes that are very rarely watched by young children. Once we had created profiles, the appropriate page/section of each platform was selected for sampling. We deliberately selected pages and carousels that were neutral in topic or style and likely to contain popular programmes (e.g. categories like “most popular” or “recommended” rather than “cartoons” or “adventure”). Where programmes were not organised into categories or pages, we sampled items from the homepage of the streaming site.

A random number generator was used to identify programmes to sample from each website. Where the programme was a series of episodes, further randomisation was used to select the exact episode to sample. If the selected programme did not meet inclusion criteria, a new programme was sampled in its place. Sampling was without replacement, but if the programme appeared in two positions on the website, it could be sampled twice. Where the randomly sampled programmes were compilations of multiple complete episodes, we selected only the first episode of the compilation to be transcribed.

The platform survey was completed on two dates. On the first date (28/03/24), 26 videos were sampled from the five platforms (enough for the first wave of data collection only). On the second date (29/07/24), a larger list of 54 randomly sampled videos was created.

### Creating video transcripts

The aim was to produce a transcript for each video sampled that contained all spoken dialogue along with part-of-speech tags for each word. Transcripts were matched as closely as possible in formatting, transcription style, and processing to CDS transcripts available through CHILDES (MacWhinney, 2000).

In total, 161 transcripts were collected between April and September 2024, with 120 transcripts (74.53%) sampled from parent survey responses and 41 (25.47%) sampled from the top five platforms. This is comparable to the Dawson et al. (2021) picture book corpus in terms of number of transcripts. Given that we did not know how many words to expect within each transcript, we did not know in advance how many transcripts would be needed. Data collection was therefore completed in four waves, in which we collected a target number of transcripts and processed each batch in its entirety before proceeding to the next batch.

#### Generating the transcripts

Pre-existing transcripts were either downloaded as.srt files or copied into a plain text file for cleaning and checking. Where no transcript was available online, the target episode was manually transcribed in Notepad by the first author (AG). We deviated from our preregistration here, since we had originally planned to use speech-to-text transcription software when no transcript was already available; however, this did not perform well when transcribing child speech or singing, and we found manual transcription to be more reliable.

Transcripts generally consisted of the entire video (including opening credits) up to the point where the end credits began, excluding advertisements. It became apparent during sampling and data collection that the videos varied significantly in length (for example, some popular YouTube channels contained compilations of episodes sometimes reaching up to 5 h in length). Including the entirety of these videos would skew our corpus towards the lexical properties of the videos that happened to be very long. We therefore deviated from our preregistration and imposed a cap on the length of the transcripts (35 min). Where videos were longer than this, we stopped the transcription at an appropriate point before this cut-off (e.g. at the end of a discrete story within episodic narratives or at the end of the last sentence before the cut-off).

#### Cleaning, checking, and editing

Prior to checking and editing, transcripts collected from pre-existing sources were cleaned in Notepad++ (v8.7.5) using regular expressions. First, all timestamps, stage directions, and speaker names were removed, and each distinct utterance was placed on a different line. The transcript was then converted to lowercase, and blank lines were removed. Every transcript was then manually checked against the original video for accuracy by the first author (AG). It was necessary to check each transcript because subtitle files and automatic transcriptions frequently contained errors or missed content. During this checking stage, transcripts were also edited to make sure they adhered to the formatting and notation principles outlined below.

#### Transcription format and notation

Transcripts were formatted according to CHAT guidelines (https://talkbank.org/manuals/CHAT.pdf) in order to match the style of the CDS data in our comparison corpus. Transcripts contain all spoken or sung content within the selected programme. Interjections (e.g. “whoa”, “ooh”, “uhoh”) are transcribed, but non-linguistic vocalisations (e.g. screaming, crying) are not. Onomatopoeias such as “woof” or “quack” are transcribed when spoken by the characters, but animal sounds produced by animal characters are not (e.g. the sound of a dog barking or a duck quacking). Similarly, while words that are sung are transcribed, non-lexical singing noises (e.g. “lalala”) are not. Spellings in transcripts matched spellings in the English MOR lexicon used by the CLAN tagging software (https://talkbank.org/). Several CHAT “special form markers” were used throughout the transcription process. These allow for the processing of forms that do not appear in the lexicon and therefore cannot be properly tagged. For example, neologisms were marked with *@n* (e.g. “cheesenado@n”) and onomateopoeias with *@o* (e.g. “woof@o”).

#### Quality control checks

At the end of each wave, after all transcripts had been collected and individually checked and edited, we conducted quality control checks across the entire set of transcripts using the search function in Notepad++. The full list of quality control checks can be found in the data collection protocol (10.17605/OSF.IO/GRBYX), but broadly this involved checking that spellings, formatting, and special form markers were consistent across all transcripts. These additional quality checks returned some inconsistencies which could then be resolved before the transcripts were processed further. For example, CHAT guidelines state that titles should be spelled out (e.g. “mister” and “doctor”), but this rule had not been applied consistently across all transcripts.

#### Part-of-speech tagging

At the end of each wave of data collection, transcripts were automatically tagged with parts of speech and parsed using the CLAN software (version 08-Sep-2023), the same software originally used to parse the comparison CDS data. The automatic morphosyntactic tagging process in CLAN (the *mor* command) works by breaking down tokens into their constituent parts using a lexicon of English morphology and a set of morphological rules. The lexicon is also used to identify a part-of-speech tag for each word (MacWhinney, 2008). Our priority was to process CCVM transcripts using the same tools that had been applied to CHILDES data, meaning that we did not use the most recent tools available for part-of-speech tagging, including within CLAN (Liu & MacWhinney, 2024). However, according to the MOR manual (MacWhinney, 2019), the English MOR grammar in CLAN (the approach chosen here) achieves 99.18% tagging accuracy for utterances from adult native speakers.

First, the plain text files were converted to.cha files using the *text2chat* function so that they could be processed with CLAN. Next, the *mor +xb* function was used to check that the words in the transcripts either appeared in the English MOR lexicon used by the programme or else were manually marked with a part of speech within the transcription. The output of this process was a list of unknown forms that could not be classified by CLAN (these were usually typing errors, uncapitalised proper nouns, British spellings, or rare forms that did not appear in the lexicon). We then edited the original text files to correct any identified errors and add tags to unknown wordforms. This process of converting, checking, and editing the transcripts was repeated until there were no more unknown forms. Once all words had been identified, the *mor* function was used to tag and parse each transcript. This resulted in a.cha file that contained the original utterances plus lexical and syntactic tagging for each word. At this point in the pipeline, the video transcripts were in the same format as the CDS transcripts (i.e. .cha files with lexical and syntactic tagging for each word).

### Processing video and CDS transcripts

From this point onwards, processing steps were applied in parallel to both the video transcripts and the subset of CDS data described above. This was done to ensure that full alignment between these corpora was possible, thereby reducing the risk of introducing arbitrary differences between the corpora that could affect later analyses. Only the CCVM data are shared in the current paper.

#### File conversion

The video and CDS transcripts were converted to .csv format so that they could be analysed in R (R Core Team, 2023) and so that the CCVM data could be shared in a format that is widely accessible by all users regardless of their specific software. This involved first converting the .cha files to .xml format then converting to .csv. The conversion to .xml was done using chatter.jar (https://talkbank.org/software/chatter.html). We then used an adapted version of an R script provided by Dawson et al. (2021) to convert the .xml files into .csv format using the XML package (Temple Lang, 2024). The output of this script was a series of .csv files containing individual tokens from the transcripts. Separate token files were combined into two large .csv files (one for video data and one for CDS data). These files contained a row for each word from each transcript, along with metadata including the source file name, corpus (“CDS” or “Video”), subcorpus (“Parent” or “Platform”), and subcorpus folder (e.g. “YouTube” or “3yos”). We removed two tokens from the CCVM at this stage that did not have an associated part of speech (both were “xxx”, a marker used to indicate unintelligible speech).

#### Manually coding lemmas

Our final processing step involved coding lemmas for each word type in our corpus. Lemmas are defined by Biber et al. (1998) as “the base form of a word, disregarding grammatical changes such as tense and plurality” (p. 29). Lemmas are helpful because they treat different variants of a word as a single group, which is useful for analyses which involve counting the number of different word types while ignoring grammatical variation within types (e.g. counting “eats”, “eating”, and “ate” as instances of the single lemma “eat”). For each word in the corpus, the CLAN software provided a part-of-speech tag and a stem form alongside the gloss (the word form exactly as it appeared in the transcript). The stem forms were generally uninflected forms of the gloss (e.g. the gloss “was” is matched to the stem “be”). However, there were a number of problems with the stem forms, which meant that they could not simply be used in place of lemmas. For example, derivational morphemes (e.g. suffixes like “-able” and “-ness” which change the grammatical category of the word) were often removed, as were parts of compound words (e.g. “babysitter” ➔ “baby”). We therefore manually coded the data to produce lemma forms in which inflectional morphemes (e.g. verb endings) were removed but derivational morphemes were preserved. To do this we identified all unique combinations of “gloss”, “stem”, and “part of speech” across our corpus and the CDS corpus and manually coded a new lemma form for each combination. We followed Bauer et al. (2013) in defining inflectional morphemes as

1. Plural and possessive marking on nouns (e.g. “cat**s**”, “father**’s**”),
2. Third-person singular marking on present-tense verbs (e.g. “rent**s**”),
3. Participle marking on verbs (e.g. “wash**ing**” and “sail**ed**”), and
4. Comparative and superlative marking on adjectives and adverbs (e.g. “happi**er**” and “clean**est**”).

All other morphemes were considered derivational and were preserved in lemma form. Any difficult cases or exceptions to these basic rules were discussed by the research team to reach a final decision. By processing the pooled video and CDS data, we ensured that lemmas were consistent between our two corpora. In total, changes were made to 3,799 stem forms across the two corpora (including 1,547 stem forms in the video corpus) to create the lemma forms.

### Metadata

During data collection, basic metadata were recorded for each transcript, as follows:

- Date of transcript collection
- Source of the transcript (e.g. YouTube, subtitle file, manual transcription)
- Programme name, episode name, and episode number
- Video length in seconds
- Country in which the programme was originally aired
- Video style (cartoon, live-action, stop-motion, or a combination of styles)
- Reality status (fiction, non-fiction, or a combination of both)
- English variety (e.g. American, British, Australian)

After compilation of the corpus, additional metadata for each programme were collected online. For each transcript, we recorded the year in which the programme was first aired. For TV series, this was the year that the particular series was first aired (using information from Wikipedia and IMDb), and for YouTube videos this was the year that the video was uploaded.

We also separately recorded age ratings, quality ratings, and genre information from Common Sense Media reviews (https://www.commonsense.org/). Common Sense Media (CSM) is a not-for-profit organisation that provides recommendations and reviews about media that is watched by children. Each review contains an overall quality rating out of 5 along with ratings for different factors (e.g. *positive role models* and *educational value*) and a minimum recommended viewing age. For each transcript, we checked to see whether there was an available review on Common Sense Media. If a match was found, we recorded the recommended minimum viewing age as well as ratings on three quality factor scales: *educational value*, *positive messages*, and *positive role models*. Since CSM is a subscription service, we have not shared review data associated with specific programmes. Instead, we have shared an anonymised dataset which details the ratings we collected without associating these with a particular programme (see *CSM-reviews.csv* on the OSF).

## Corpus contents and structure

The corpus contains 233,471 tokens and 10,545 distinct word types (distinct gloss forms) over a total of 161 transcripts. The corpus includes transcriptions of 43.12 h of video (31.06 h in the parent subcorpus and 12.07 h in the platform subcorpus). In the following sections we describe the content of the corpus in terms of (a) the proportions of transcripts from each source and their basic properties, and (b) the characteristics of the included programmes.

### Sampling proportions and transcript properties

#### Transcripts

**Fig. 3:**
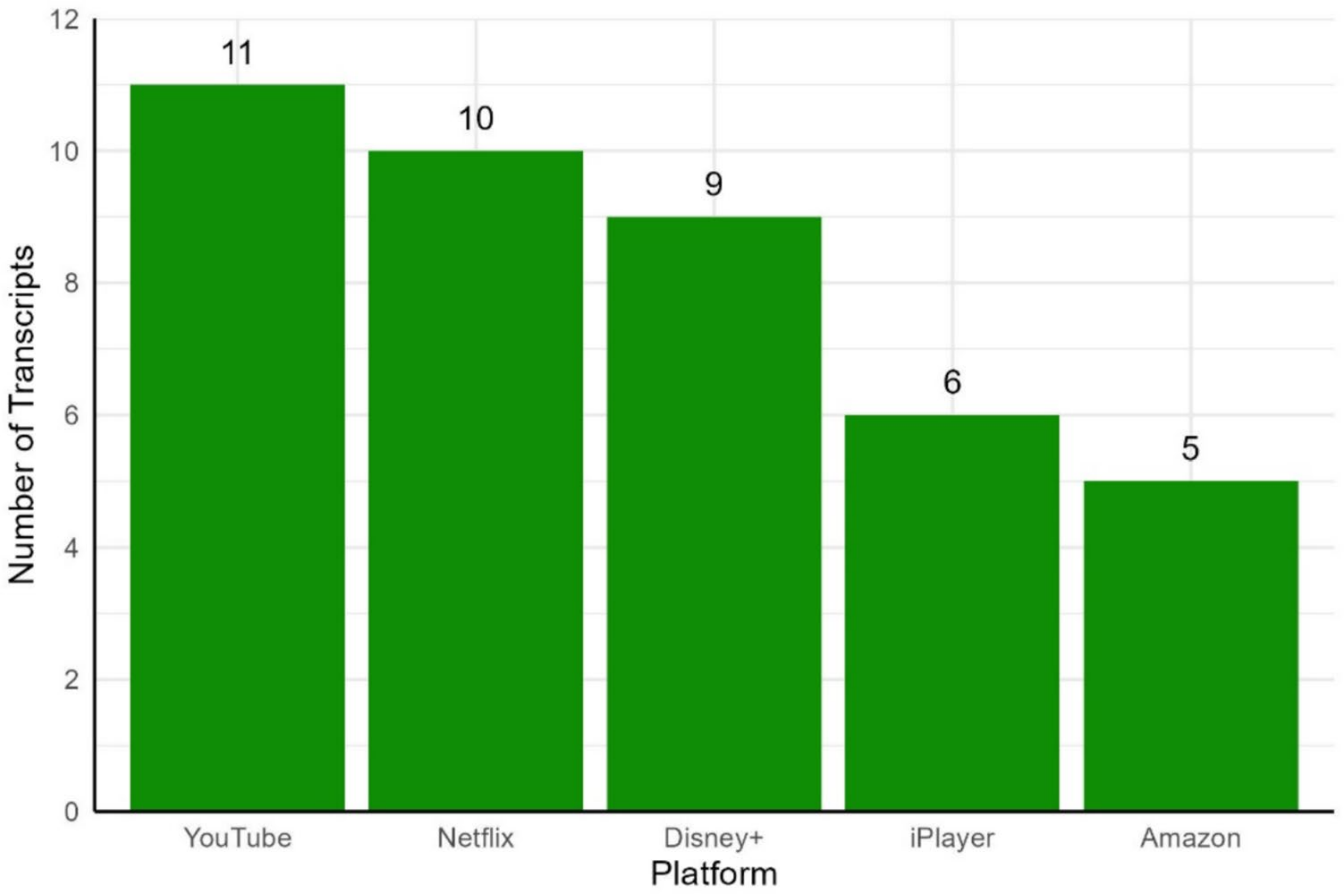
Number of transcripts within the platform subcorpus sampled from each of the five most popular platforms in our parent survey

Among the included transcripts, 120 (75.53%) were sampled from the parent answers, and 41 (25.47%) were sampled from platforms. Within the parent subcorpus, transcripts were balanced across our age range (40 transcripts each for 3-, 4-, and 5-year-olds). The distribution of transcripts in the platform subcorpus was determined by the popularity of each platform in the parent survey (see Fig. 3).

#### Tokens

**Fig. 4:**
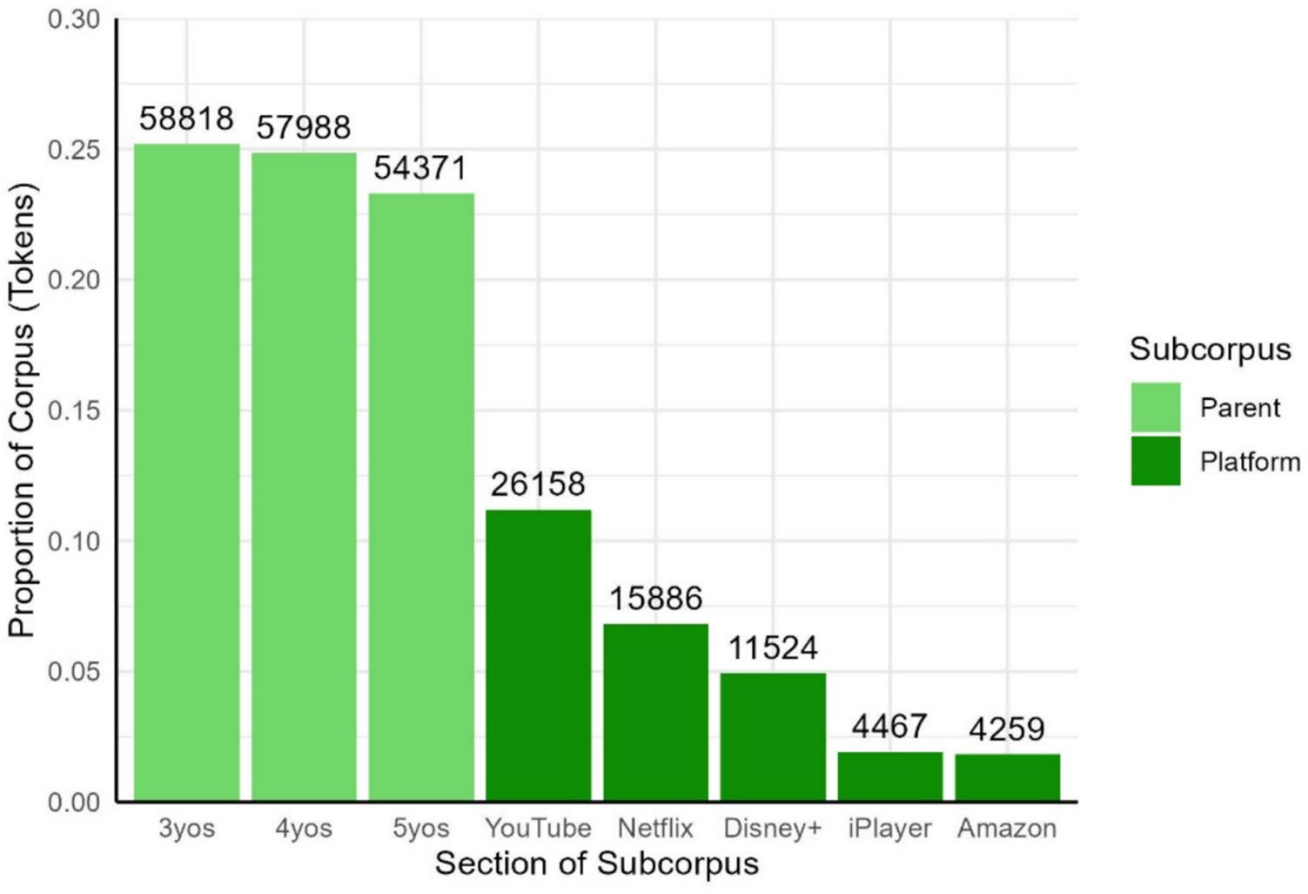
Proportion of tokens from each section of each subcorpus. The *y*-axis indicates the proportion of the subcorpus made up by each age band and platform, while the numbers above the bars indicate numbers of tokens

Among the tokens in the corpus, 73.32% come from the parent subcorpus (171,177 tokens, 8,919 types) and 26.68% from the platform subcorpus (62,294 tokens, 4,837 types). Within the parent subcorpus, tokens were roughly evenly divided between the three age bands, while for the platform corpus, the proportion of words from each source did not correspond directly to the proportion of transcripts (Fig. 4). This difference arises due to differences in video length and speech rate for different platforms.

#### Video length and speech rate

**Fig. 5:**
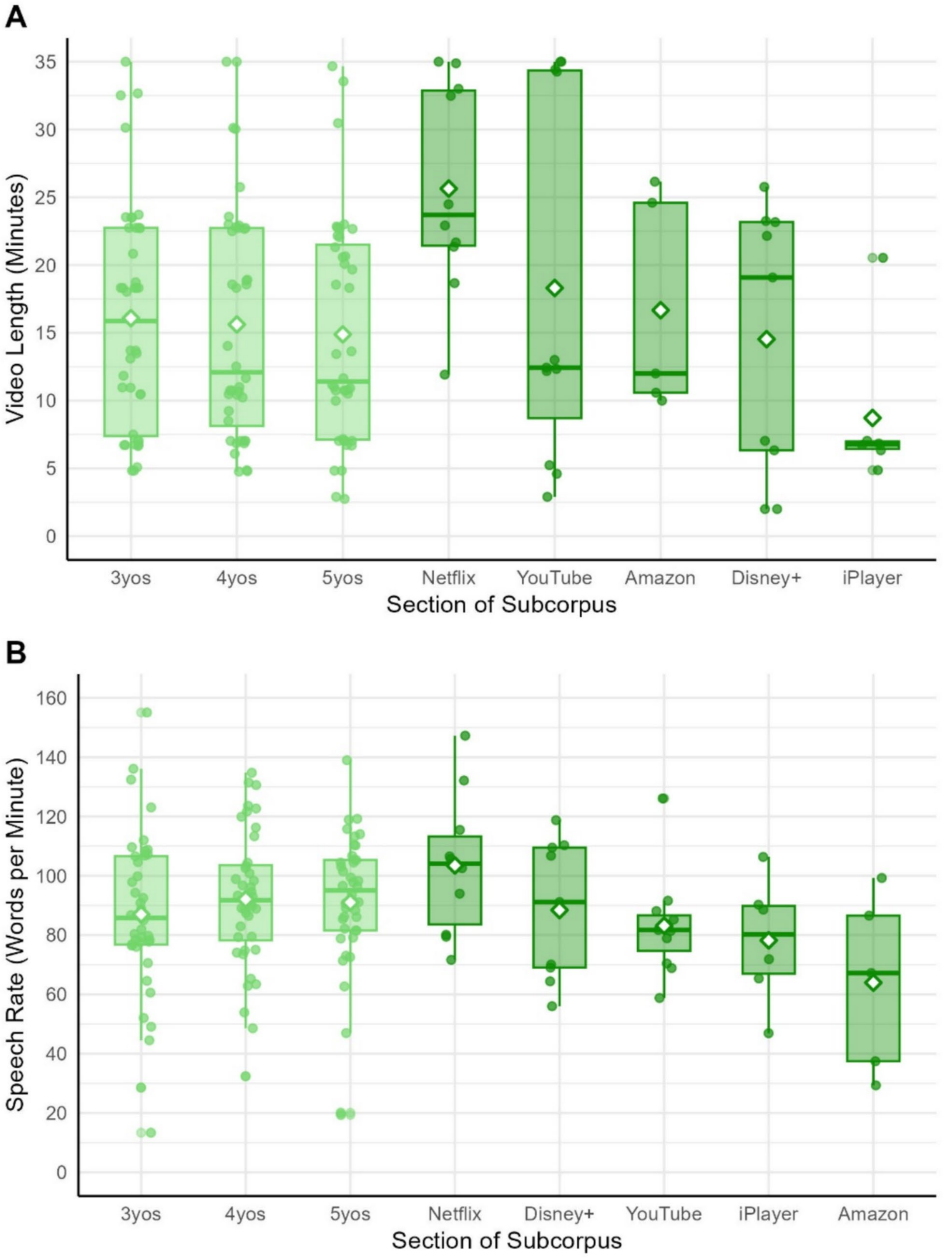
**A** Video length in minutes split by subsection within each subcorpus. Green points indicate individual transcripts, while white diamonds indicate the mean length for each subsection of the corpus. **B** Number of words per minute split by subsection within each subcorpus. Green points indicate individual transcripts, while white diamonds indicate the mean speech rate for each subsection of the corpus

On average, videos lasted for 16.07 min (*SD* = 9.13 min). Videos ranged from 2 to 35 min, and length was highly variable within all age bands and platforms (Fig. 5a). The upper end was determined by our 35-min cut-off. In reality, videos available to children on streaming platforms frequently last for 1 h or longer (with some videos lasting up to 5 h). The average speech rate across the whole corpus is 90.23 words per minute (Fig. 5b). The differences in speech rate and video length across platforms may indicate different norms and approaches to children’s media on each platform. For example, many of the programmes on Netflix are either longer narrative stories (e.g. *Barbie Dreamhouse Adventure*) or long compilations of short songs (e.g. *Cocomelon*). By contrast, programmes sampled from iPlayer are much shorter narratives or short videos intended to teach simple concepts or words (e.g. *Colourblocks*). Variability in these simple transcript properties suggests our corpus captures a broad range of examples of children’s video media.

### Programme characteristics

#### Year

The programmes in the corpus were released over a 31-year period between 1993 and 2024. The majority of programmes (*n* = 121, 75.16%) were first released within the last 10 years. Recently released programmes are most often available and promoted on streaming platforms.

#### Country and variety of English

Most programmes were created in the UK or the USA, but 10 countries were represented (Table 2). Included programmes used three English varieties, with British English being the most common (Table 3).

**Table 2:** Country in which programmes were originally aired

Country	No. transcripts	Proportion
UK	71	0.44
USA	54	0.34
Australia	15	0.09
Canada	12	0.07
France	3	0.02
South Korea	2	0.01
Argentina	1	0.01
Israel	1	0.01
Japan	1	0.01
Russia	1	0.01

**Table 3:** Primary variety of English used in each transcript

English variety	No. transcripts	Proportion
British	85	0.53
American	61	0.38
Australian	15	0.09

#### Visual style

Programmes were coded according to their visual style (live-action, cartoon, stop-motion, or a combination of styles). The majority of programmes were cartoons (73%), as can be seen in Table 4.

**Table 4:** Visual style of videos that transcripts were based on

Style	No. videos	Proportion
Cartoon	117	0.73
Live-action	38	0.24
Combination	4	0.02
Stop-motion	2	0.01

#### Expert reviews

**Fig. 6:**
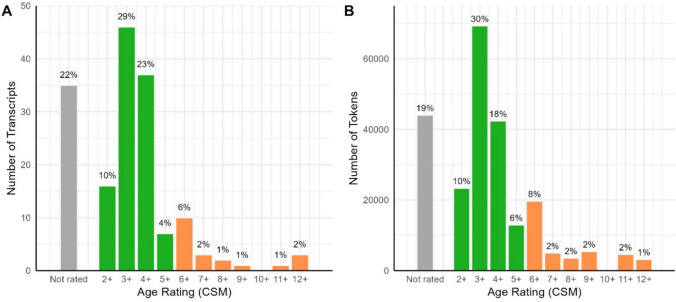
Proportion of transcripts (**A**) and associated tokens (**B**) in the corpus receiving each age rating from Common Sense Media. In both panels, the *x*-axis indicates the rating provided by CSM, the *y*-axis indicates raw transcript or token counts, bar labels indicate percentage of the total number of transcripts/tokens receiving that rating, and colour indicates rating category (grey = not rated; green = rated as appropriate for target age group; orange = rated as appropriate for children over *5*)

Age rating data were taken from expert reviews on Common Sense Media. No data were available for 35 transcripts across 25 programmes (21.74% of the transcripts). Appropriate age was rated on a scale from 2+ to 17+ years, taking into consideration both positive content (e.g. “positive role models”) and negative content (e.g. “violence & scariness”). Overall age ratings reflect positive and negative content and developmental guidelines. Ratings of programmes in this corpus ranged from 2+ to 12+ years (Fig. 6). Although most of the reviewed programmes were deemed suitable for the target age range (84.13% rated 5+ or lower), 15.87% of reviewed programmes were rated 6+ or higher, indicating that they were inappropriate for children in our target age range. The programmes with the highest age ratings include *The Simpsons* (12+) and *Mr Bean: The Animated Series* (12+). Of the programmes that were rated as inappropriate, 15 out of 20 were drawn from the parent survey.

**Fig. 7:**
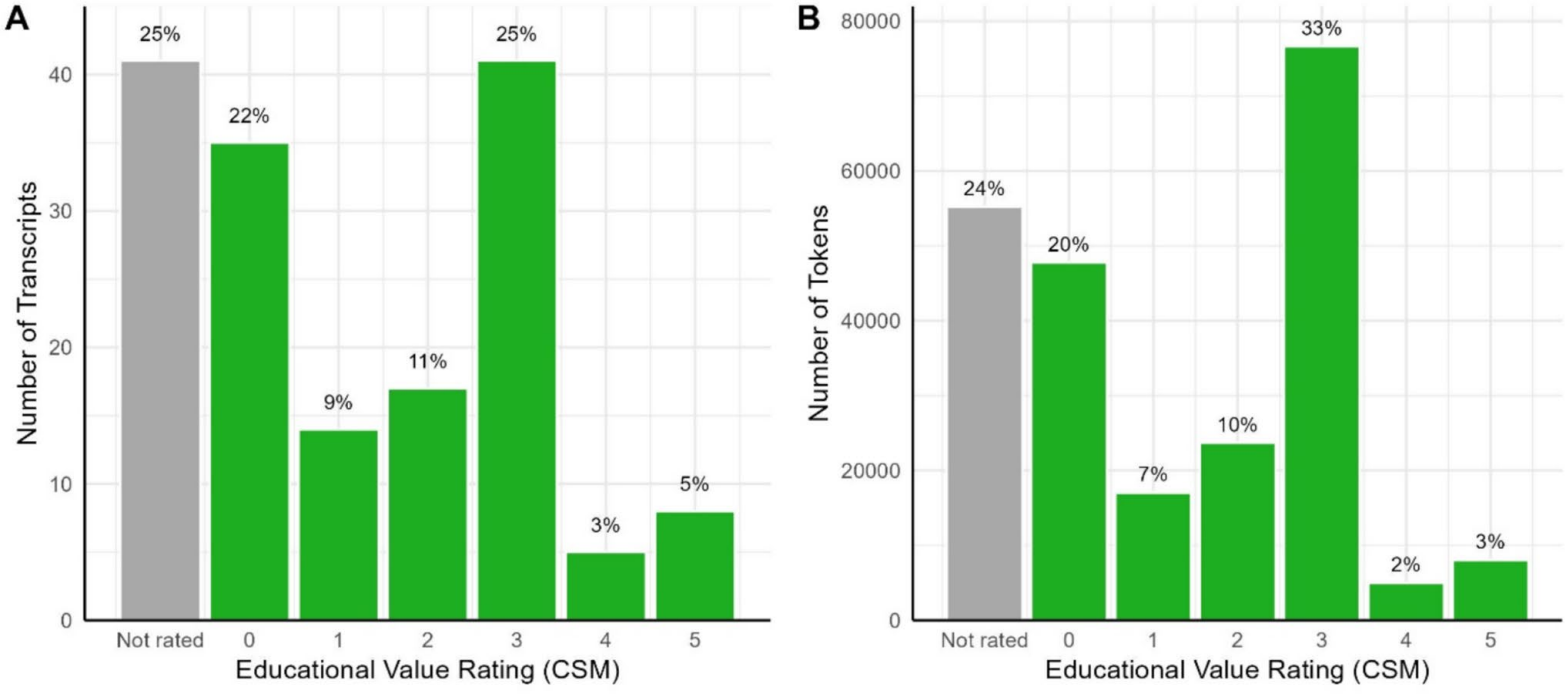
Proportion of transcripts (**A**) and associated tokens (**B**) in the corpus receiving each educational value rating from Common Sense Media

Educational value ratings from Common Sense Media were available for 120 transcripts (Fig. 7). This scale (0–5) considers whether a programme educates children, whether this is intentional or incidental, and what specific content is taught (https://www.commonsensemedia.org/about-us/our-mission/about-our-ratings). While the most common rating was 3 out of 5, a large proportion of transcripts received a rating of 0, meaning that either the programme was judged to have no educational value, or the educational value scale was not applicable to that programme. Only a small number of transcripts received the top rating of 5 (e.g. *The Octonauts* and *Numberblocks*).

Overall, the available reviews suggest that our sample (selected for programme popularity and availability for young children) includes both educational and entertainment programmes that are not always rated as being appropriate for young children. While CSM ratings are a useful way to characterise the programmes in our sample using metrics that are independent of both content producers and individual families, these ratings are based on principles of what is generally appropriate for a certain developmental stage and do not necessarily reflect what is appropriate for an individual child. Parents likely have a better understanding of what is appropriate for their own child’s developmental level and may therefore allow their child to watch programmes that have been given a higher age rating on CSM (for evidence that parents give lower age ratings, see Feng & Zhu, 2022). Importantly, the disparity between parent choices and expert reviews suggests that a corpus that only included “age-appropriate” programmes according to reviews would not be representative of the programmes that children are actually watching.

#### Specific programmes included

**Fig. 8:**
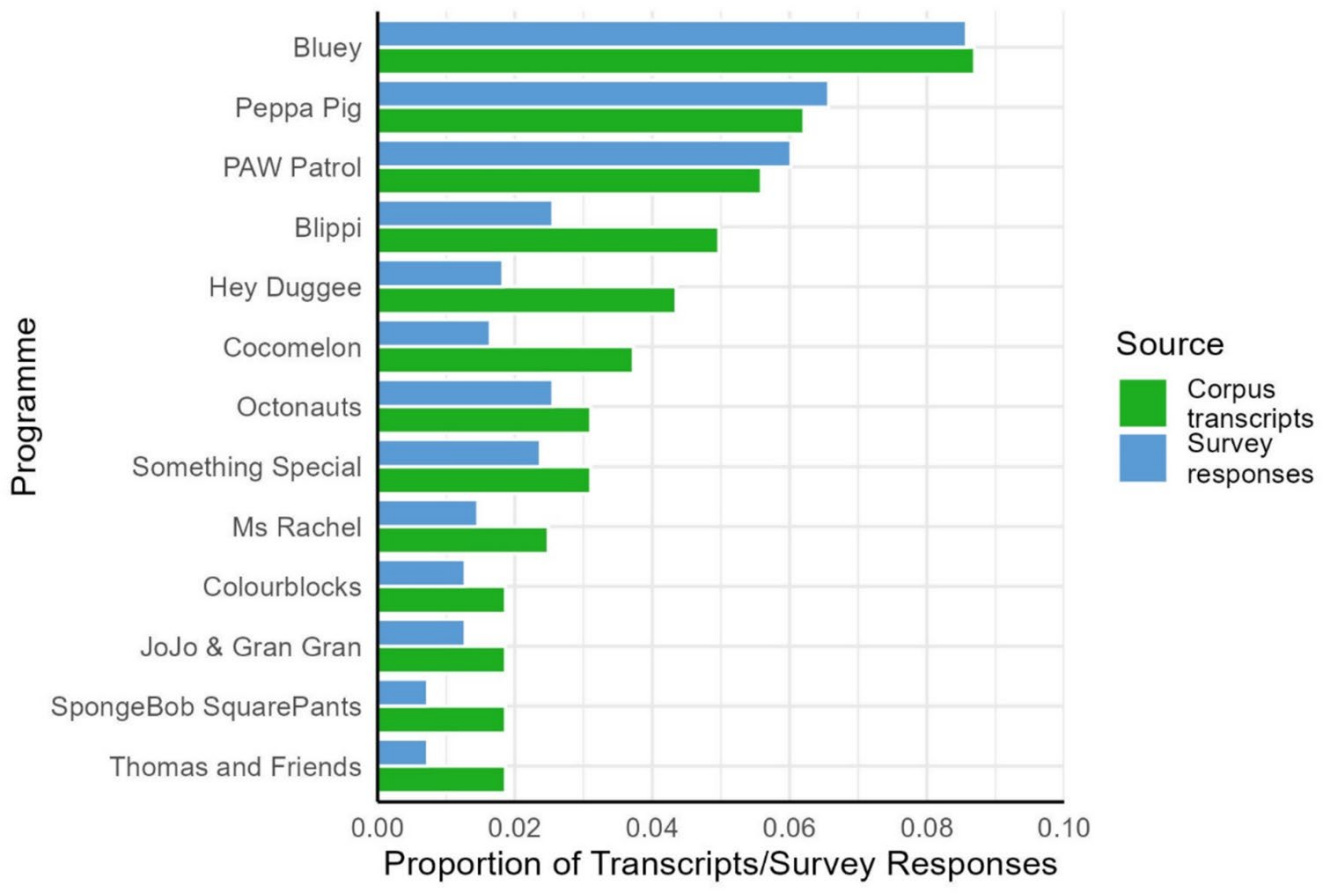
Proportion of transcripts in the corpus belonging to a particular programme (green bars) and proportion of parent responses matching particular programmes (blue bars). Graph includes all programmes that appear more than twice in the corpus

The corpus contains transcripts from 84 different programmes. We did not restrict the number of times a particular programme could appear in the corpus. As a result, programmes that were more popular (according to our parent survey) or more readily available (according to our platform survey) appear multiple times. The number of transcripts from a particular programme ranged from 1 (e.g. *Batwheels*) to 14 (*Bluey*). In total, 61 programmes appear only once in the corpus. Figure 8 shows the programmes that are represented the most in the corpus and how this compares to the responses from the parent survey. The proportion of transcripts in the corpus for each programme is closely matched to the number of times that programme was mentioned by parents.

**Fig. 9:**
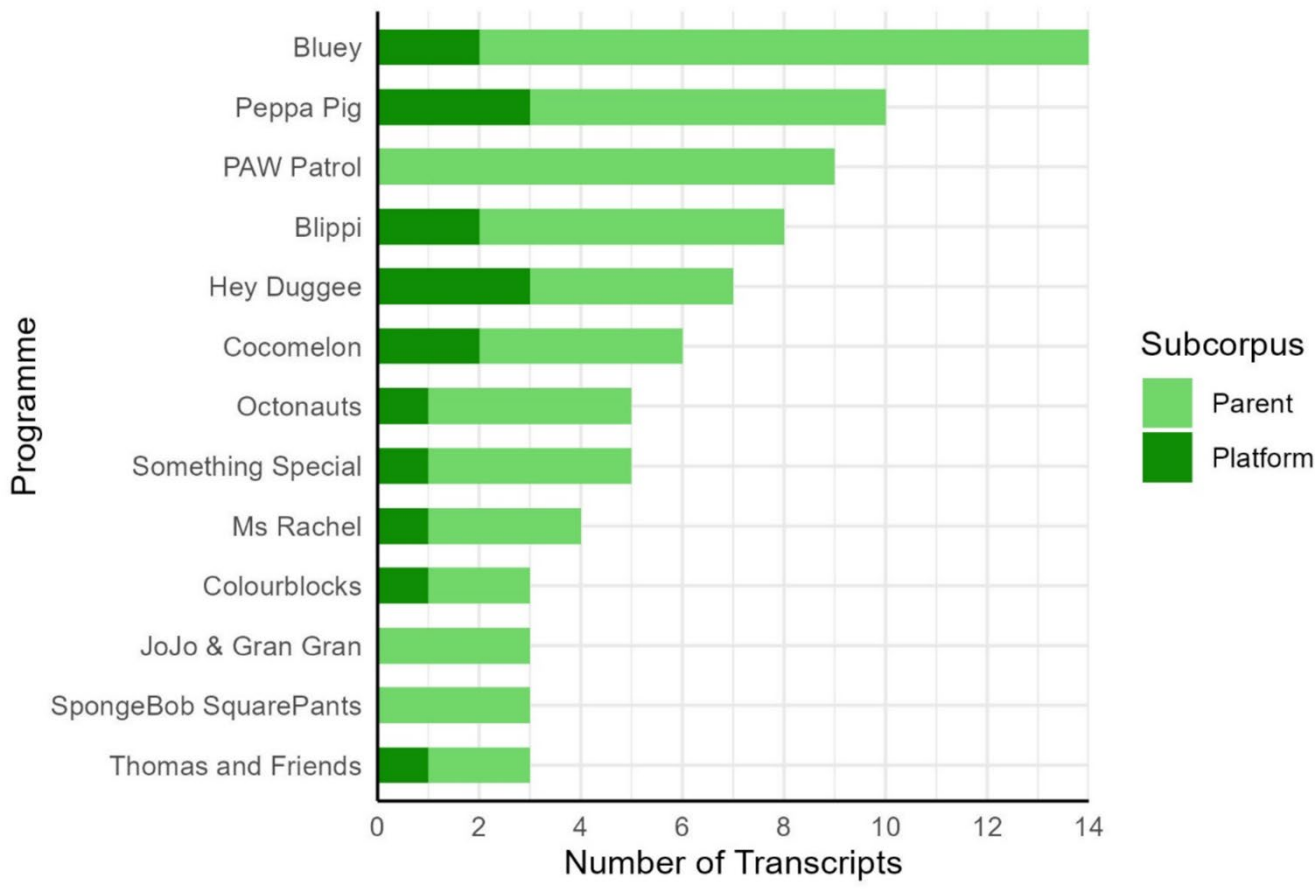
Number of transcripts belonging to particular programmes split by subcorpus. *Grap*h includes all programmes that appear more than twice in the corpus

There are some programmes which have been oversampled relative to the proportion of parent responses (e.g. *Blippi* and *Cocomelon*). Looking at Fig. 9, we can see that in most cases this is because the additional transcripts have been collected as part of our platform survey. In total, 23 of the programmes in the platform subcorpus never appeared in the parent subcorpus (e.g. *Little Angel*, *Postman Pat*, *Super Kitties*). These could be programmes that are less salient to parents or less socially desirable and therefore less likely to be reported. These patterns suggest that our dual sampling technique has successfully captured patterns of programme popularity among parents while also supplementing our corpus with a distinct set of programmes that are readily available on streaming platforms. The distribution of programmes within the corpus therefore represents patterns of popularity and availability: a small number of programmes are mentioned by a large proportion of parents and appear on multiple platforms, while a much larger set of titles are more obscure in that they were mentioned only a handful of times across our parent sample or they only appeared on a single platform. However, it is not clear from the present data whether this distribution of titles is also reflective of the viewing habits of individual children. Further research is needed to understand whether children prefer to watch their favourite titles repeatedly, or whether their viewing experience is more varied.

## Discussion

This paper describes the creation and contents of the Corpus of Children’s Video Media, a specialised corpus of videos popular among 3–5-year-olds in the UK. Many of our design decisions were guided by our intention to compare the present corpus to a CDS corpus. We have followed open science practices throughout the process, including preregistering aspects of our study (sampling, data collection procedures, hypotheses), describing our sampling procedure in detail, and making the corpus available on the OSF as a series of tokens matched to part-of-speech tags, scrambled within each transcript (10.17605/OSF.IO/GRBYX). We have also shared additional materials including the metadata for each transcript, R scripts to reproduce our figures and analysis, and a detailed protocol for data collection intended to provide a useful example for other researchers looking to build upon the CCVM or create their own specialised corpus.

### Strengths of the corpus

One of the strengths of this corpus is its specificity. The care taken in sampling episodes and videos to include in the corpus means that it provides a precise window into the language that children are exposed to through video media, in contrast to other, larger corpora with less specific inclusion criteria. Although reliability is often simplistically linked to corpus size, Koester (2010) argues that specialised corpora can be used to obtain reliable results at a smaller sample size, because they can represent the target language domain better than larger and more general corpora. We have also tried to ensure that the corpus is representative of our target domain (programmes watched by 3–5-year-olds in the UK) by sampling from a variety of sources. The platform survey allowed us to include a broader range of titles than if we had relied on parent answers alone, increasing the variety of programme types in our sample. This variety, alongside our focus on popular and readily available programmes, increases the representativeness of our corpus.

The relatively small size of our corpus also allows for greater quality control for included transcripts. While it would be possible to increase the size of the corpus by including unchecked subtitle files or artificial intelligence (AI)-generated transcripts, we observed a great number of inaccuracies in these data sources that could potentially impact study results. This conclusion echoes Bednarek (2020), who created a corpus of 66 TV episodes in which 70% of transcripts were manually produced. Bednarek also notes that manually transcribing programmes allows researchers to include programmes for which there is no pre-existing transcript available online.

Finally, since we have shared our data, code, and data collection protocol, it should be straightforward for other researchers to build upon our work, for example by extending the corpus to include more transcripts, adding additional annotation, or analysing a subset of the data. This transparency also allows other researchers to evaluate how well the corpus represents our target domain of programmes watched by 3–5-year-olds in the UK. The CCVM data available on the OSF could also be converted to a lexical database similar in format to SUBTLEX-UK (van Heuven et al., 2014) or CYP-LEX (Korochkina et al., 2024) containing summary information for each word form (e.g. frequency, part-of-speech, lexical diversity). A lexicon of this kind would facilitate comparisons with existing databases (e.g. CBP-LEX, which contains language data from children’s picture books; Green et al., 2023), thereby supporting additional research questions. It would also increase usability of the corpus for researchers with limited coding skills. Note that additional data processing steps would be required to ensure comparability with existing lexical databases, as these are likely to have followed different transcription principles.

### Limitations of the corpus

The corpus is relatively small, with only 233,471 tokens, and is therefore unsuitable for studies of low-frequency phenomena (e.g. the usage of particularly rare words or phrases). The relatively small size of the corpus also means that we could not capture the full diversity of the viewing experience of our target group. In particular, we focus primarily on professionally produced programmes available from streaming sites rather than amateur short-form content on platforms like YouTube and TikTok. Since short-form content is increasingly popular among children (Ofcom, 2024), future work should explore the linguistic properties of these videos.

Our corpus necessarily represents a snapshot of children’s viewing habits in 2024. Although some of the included videos have been popular for a long time now (e.g. *Peppa Pig* was first aired in 2004), viewing habits can change rapidly, and our corpus may not be representative of children’s viewing in a few years’ time. This is more challenging for video-based corpora than, say, books or CDS, since the technology for viewing and interacting with video media is constantly changing, and so viewing habits are less likely to be stable. For example, Ofcom (2024) reported a 14% drop in the amount of time 4–15-year-olds spent viewing live broadcast TV via TV sets from 2022 to 2023.

The corpus is necessarily restricted by the type of information we captured in our transcripts. The transcripts are comprehensive in terms of spoken and sung dialogue and conversational markers, since this is what was required to analyse the lexical properties of children’s video media. However, we do not (yet) include phonetic transcriptions, speaker names, or information about visual elements of the programmes. The corpus in its current form is therefore inappropriate for analyses of accent, dialogue, or visual cues. However, since we have shared the programme titles and episode numbers for each transcript, future researchers will be able to add additional coding to the corpus.

Finally, although we have taken steps to try to avoid bias (i.e. preregistering our sampling plan, collecting data from parents, randomly sampling where possible), it was impossible to remove all risk of bias in our procedures for programme selection. For example, our sample might not capture the viewing experiences of children from low-SES backgrounds who may not have access to subscription streaming services like Netflix and Disney+. However, this issue primarily affects programmes which are only available from one source, and most of the top programmes in our sample are available from a number of sources, including live TV and YouTube. Similarly, there is also likely an SES bias in our comparison CDS corpus since the studies that contributed the most data to our corpus describe their samples as “middle-class” (Lieven et al., 2009) or state that the target child’s parents were university graduates (Rowland & Fletcher, 2006).

### Comparability with CDS corpus

In order to make valid inferences about the differences between video language and CDS, it is important that the two corpora are comparable and do not differ in arbitrary ways that are irrelevant to the research question. We have tried to ensure this comparability by matching the corpora on important design features such as target age range and transcription style, as well as applying the same data processing steps to both corpora. However, it was not possible to match the corpora in every aspect, and some important limits to their comparability are as follows:

- Transcription style: Although we followed the same transcription guidelines as the CDS corpus (CHAT format), there are still some inconsistencies in how some speech features are transcribed. Since the CDS corpus is compiled using data from multiple studies, there are transcription inconsistencies within this corpus. For example, the CDS corpus contains multiple spellings of some words (e.g. “ok” vs “okay”), making it impossible for our corpus to be fully consistent. In addition, the fact that transcripts were created by different people (both between the CDS corpus and the CCVM and within the CDS corpus) means that the more subjective elements of the transcriptions are likely to vary (e.g. the decision to transcribe an interjection as “ah” vs “ahh”).
- Date range: The CDS corpus contains data collected between 1973 and 2008, while the video corpus is entirely composed of videos available online in 2024. This large discrepancy in dates is unfortunate but not easily avoided given the lack of publicly available CDS data in recent years.
- Corpus size: Since it was not feasible to match the CCVM to the CDS corpus in terms of token count or transcript count, the CDS corpus is significantly larger than the CCVM. As such, any comparison of the two corpora must use methods that are robust to differences in corpus size (e.g. random sampling approaches or relative frequencies). Comparisons of low-frequency phenomena between the two corpora will likely be unreliable, even when adjusted for corpus size.

These limits to comparability must be taken into account when analysing the data and interpreting the differences observed between the two corpora. For example, given that we know transcriptions of interjections (e.g. “ah”, “ooh”) are likely to be unreliable, we might choose to exclude these from the analysis. This further highlights the importance of sharing clear documentation about how corpora were compiled, since transparency about the differences between corpora is crucial to evaluating the validity of any corpus comparison study.

In addition to technical differences between corpora, there are also inherent differences between sources of language input that are difficult to capture in corpora. For example, the rate at which children encounter words may differ across conversations versus books and videos, and children may spend different amounts of time engaged in each activity. One way to resolve these difficulties is through modelling studies which estimate the amount of time or exposure needed to learn new words or linguistic structures from a particular language source (e.g. Nation, 2014; Green, 2021; Green & Sun, 2024).

### Corpus construction advice

There were two key steps that were particularly helpful in setting up this project: preregistration and piloting. Preregistration is increasingly common in experimental psychology but is not a practice that has been widely adopted in corpus linguistics (Mak, 2024). There are many researcher degrees of freedom in corpus linguistics, and preregistration is a helpful way to limit the methodological choices in advance of seeing the data. This has the result of placing a focus on a priori hypotheses and reducing opportunities for selective reporting, thereby increasing scientific rigour and minimising bias at the analysis stage. It may not always be possible to preregister a full analysis plan for a corpus study, because the final size and format of the dataset is not always known in advance (for a discussion see Mak, 2024). However, we strongly recommend that researchers at least preregister their hypotheses, sampling plan, and data collection pipeline, both because it reduces the risk of confirmation bias and because it helpfully constrains what can otherwise be an overwhelming and open-ended process.

Prior to preregistering our study, we also piloted our data collection pipeline from the initial transcript creation to preparing the data for analysis. This process was incredibly helpful both for estimating the time it would take to create the final corpus and for understanding the formatting requirements for each processing stage. Before beginning data collection, we would encourage researchers to collect 5–10 transcripts and run them through the entire data collection pipeline. There will almost certainly be unexpected technical problems along the way, and it is better to identify these at the start of your project.

## Conclusions

The Corpus of Children’s Video Media, a specialised corpus of TV shows and videos popular among children, is available to other researchers through the OSF as a scrambled database of tokens (10.17605/OSF.IO/GRBYX). This corpus was built to be comparable to a corpus of CDS, but our methods also make possible comparisons with other corpora and allow for future extensions of our corpus to include a wider range of ages, video programming, and research questions. We have provided examples of key decisions involved in creating a corpus for a specific research project, to benefit researchers who are new to corpus linguistics and those looking to make cross-corpora comparisons. We have discussed the challenges involved in creating a corpus that is well matched to a pre-existing corpus created by a different team for different purposes and have provided examples of the specific difficulties we faced and the data processing steps required to harmonise the two corpora. We have highlighted the importance of transparent documentation in facilitating cross-corpora comparisons and have, therefore, provided extensive open materials outlining all the steps we used to get from the raw transcripts to the final database. Since much of the process involves bespoke code or specialised programmes not used outside of corpus linguistics, we hope that these instructions will save researchers time as well as allowing more meaningful cross-corpora comparisons.

## Data Availability

The datasets generated during and analysed during the current study are available in the Open Science Framework repository, 10.17605/OSF.IO/GRBYX

## Footnotes

1 We excluded studies if the target child was bilingual or had a developmental diagnosis (*N* = 2), if the data were in an inappropriate format (*N* = 4), if the conversation setting was highly structured (*N* = 2), or if the target children were younger than 3 or older than 5 (*N* = 6). Within the eligible studies (*N* = 8), we excluded any transcripts with missing data or errors in the file (*N* = 18), files that were in the wrong format (*N* = 59), or files in which the target child was not aged 3–5 years (*N* = 1,265). This left us with a total of 734 files from eight different studies to include in the comparison corpus (a list of included studies can be found in Appendix I). We also filtered out speech from the target child and tokens with missing part-of-speech data.

2 We deviated from our preregistration by also excluding “vlogs”, which simply follow the daily lives of the subjects. This meant that YouTube channels such as Ms Rachel (a popular infant-directed channel) were included, while channels documenting the lives of children (e.g. Kids Diana Show) were excluded.

## Code availability

Analysis code is available on the Open Science Framework: 10.17605/OSF.IO/GRBYX

## References

[CR1] Ädel, A. (2020). Corpus Compilation. In M. Paquot & STh. Gries (Eds.), *A practical handbook of corpus linguistics* (pp. 3–24). Springer International Publishing. 10.1007/978-3-030-46216-1_1

[CR2] Andersen, G. (2016). Semi-lexical features in corpus transcription: Consistency, comparability, standardisation. *International Journal of Corpus Linguistics,**21*(3), 323–347. 10.1075/ijcl.21.3.02and

[CR3] Anderson, N. J., Graham, S. A., Prime, H., Jenkins, J. M., & Madigan, S. (2021). Linking quality and quantity of parental linguistic input to child language skills: A meta-analysis. *Child Development,**92*(2), 484–501. 10.1111/cdev.13508 33521953 10.1111/cdev.13508

[CR4] Anwyl-Irvine, A. L., Massonnié, J., Flitton, A., Kirkham, N., & Evershed, J. K. (2020). Gorilla in our midst: An online behavioral experiment builder. *Behavior Research Methods,**52*(1), 388–407. 10.3758/s13428-019-01237-x 31016684 10.3758/s13428-019-01237-x PMC7005094

[CR5] Bauer, L., Lieber, R., & Plag, I. (2013). *The Oxford Reference Guide to English Morphology* (1st ed). Oxford University Press.

[CR6] Bednarek, M. (2020). The Sydney corpus of television dialogue: Designing and building a corpus of dialogue from US TV series. *Corpora,**15*(1), 107–119. 10.3366/cor.2020.0187

[CR7] Biber, D., Reppen, R., & Conrad, S. (1998). *Corpus Linguistics: Investigating Language Structure and Use*. Cambridge University Press. 10.1017/CBO9780511804489.003

[CR8] Biemiller, A., & Boote, C. (2006). An effective method for building meaning vocabulary in primary grades. *Journal of Educational Psychology,**98*(1), 44–62. 10.1037/0022-0663.98.1.44

[CR9] Brodsky, W., & Sulkin, I. (2021). What babies, infants, and toddlers hear on Fox/Disney BabyTV: An exploratory study. *Psychology of Popular Media,**10*(3), 330–339. 10.1037/ppm0000321

[CR10] Davies, M. (2021). The TV and movies corpora: Design, construction, and use. *International Journal of Corpus Linguistics,**26*(1), 10–37. 10.1075/ijcl.00035.dav

[CR11] Dawson, N., Hsiao, Y., Tan, A., Banerji, N., & Nation, K. (2021). Features of lexical richness in children’s books: Comparisons with child-directed speech. *Language Development Research,**1*(1), 9–53. 10.34842/5WE1-YK94

[CR12] Dowdall, N., Melendez-Torres, G. J., Murray, L., Gardner, F., Hartford, L., & Cooper, P. J. (2020). Shared picture book reading interventions for child language development: A systematic review and meta-analysis. *Child Development,**91*(2), e383–e399. 10.1111/cdev.13225 30737957 10.1111/cdev.13225

[CR13] Egbert, J., Biber, D., & Gray, B. (2022). *Designing and Evaluating Language Corpora: A Practical Framework for Corpus Representativeness*. Cambridge University Press. 10.1017/9781316584880

[CR14] Farrant, B. M., & Zubrick, S. R. (2012). Early vocabulary development: The importance of joint attention and parent-child book reading. *First Language,**32*(3), 343–364. 10.1177/0142723711422626

[CR15] Feng, G. C., & Zhu, S. (2022). Dynamics of rater differences in assessing the age appropriateness of media content: A multilevel moderated mediation analysis. *Journal of Broadcasting & Electronic Media,**66*(1), 68–88. 10.1080/08838151.2021.1964502

[CR16] Gathercole, V. C. (1986). The acquisition of the present perfect: Explaining differences in the speech of Scottish and American children. *Journal of Child Language,**13*(3), 537–560. 10.1017/S0305000900006875 3793814 10.1017/s0305000900006875

[CR17] Green, C. (2021). Extensive viewing of children’s entertainment and the potential for incidental learning of early years reading vocabulary: A corpus study. *Language and Education*. 10.1080/09500782.2021.1983587

[CR18] Green, C., & Sun, H. (2024). Picturebooks increase the frequency and diversity of emotion vocabulary in children’s language environments: Modeling potential benefits to emotional literacy, with pedagogical resources. *Early Education and Development*. 10.1080/10409289.2024.2423259

[CR19] Green, C., Keogh, K., Sun, H., & O’Brien, B. (2023). The children’s picture books lexicon (CPB-Lex): A large-scale lexical database from children’s picture books. *Behavior Research Methods,**56*, 4505–4521. 10.3758/s13428-023-02198-y 10.3758/s13428-023-02198-y PMC11289352 37566336

[CR20] Hartmann, S. (2024). Open Corpus Linguistics – or How to overcome common problems in dealing with corpus data by adopting open research practices. In M. Kaunisto & M. Schilk (Eds.), *Challenges in Corpus Linguistics* (pp. 89–105). John Benjamins. https://www.jbe-platform.com/content/books/9789027246530-scl.118.06har. Accessed 15 Apr 2025.

[CR21] Jing, M., Ye, T., Kirkorian, H. L., & Mares, M.-L. (2023). Screen media exposure and young children’s vocabulary learning and development: A meta-analysis. *Child Development,**94*(5), 1–21. 10.1111/cdev.13927 10.1111/cdev.13927 37042116

[CR22] Kaefer, T., Neuman, S. B., & Pinkham, A. M. (2025). Designing children’s media: Taxonomies as a scaffold for learning and attention. *Media Psychology*. 10.1080/15213269.2025.2454269

[CR23] Koester, A. (2010). Building small specialised corpora. In M. A. O’Keeffe & J. McCarthy (Eds.), *The Routledge Handbook of Corpus Linguistics* (1st ed., pp. 66–79). Routledge.

[CR24] Korochkina, M., Marelli, M., Brysbaert, M., & Rastle, K. (2024). The children and young people’s books lexicon (CYP-LEX): A large-scale lexical database of books read by children and young people in the United Kingdom. *Quarterly Journal of Experimental Psychology,**77*(12), 2418–2438. 10.1177/17470218241229694 10.1177/17470218241229694 PMC11607844 38262912

[CR25] Lieven, E., Salomo, D., & Tomasello, M. (2009). Two-year-old children’s production of multiword utterances: A usage-based analysis. *Cognitive Linguistics,**20*(3), 481–507. 10.1515/COGL.2009.022

[CR26] Liu, H., & MacWhinney, B. (2024). Morphosyntactic analysis for CHILDES. *Language Development Research*, *4(1).*10.34842/j97r-n823

[CR27] MacWhinney, B. (2000). *The CHILDES Project: Tools for analyzing talk* (3rd ed). Lawrence Erlbaum Associates. 10.1162/coli.2000.26.4.657

[CR28] MacWhinney, B. (2008). Enriching CHILDES for morphosyntactic analysis. *Carnegie Mellon University. *10.1184/R1/6614687.v1

[CR29] MacWhinney, B. (2019). MOR Manual. *Carnegie Mellon University*. 10.21415/T5B97X. Retrieved from https://web.archive.org/web/20200210174250id_/https://talkbank.org/manuals/MOR.pdf. Accessed 28 Oct 2025.

[CR30] Mak, M. H. C. (2024). Corpus linguistics will benefit from greater adoption of pre-registration: A novice-friendly split-corpus approach to pre-registration. *Applied Corpus Linguistics,**4*(3), Article 100111. 10.1016/j.acorp.2024.100111

[CR31] Montag, J. L. (2019). Differences in sentence complexity in the text of children’s picture books and child-directed speech. *First Language,**39*(5), 527–546. 10.1177/0142723719849996 31564759 10.1177/0142723719849996 PMC6764450

[CR32] Montag, J. L., Jones, M. N., & Smith, L. B. (2015). The words children hear: Picture books and the statistics for language learning. *Psychological Science,**26*(9), 1489–1496. 10.1177/0956797615594361 26243292 10.1177/0956797615594361 PMC4567506

[CR33] Nation, P. (2014). How much input do you need to learn the most frequent 9,000 words? *Reading in a Foreign Language,**26*(2), 1–16. 10.26686/wgtn.12543437.v1

[CR34] Nation, K., & Snowling, M. J. (2004). Beyond phonological skills: Broader language skills contribute to the development of reading. *Journal of Research in Reading,**27*(4), 342–356. 10.1111/j.1467-9817.2004.00238.x

[CR35] Nation, K., Dawson, N. J., & Hsiao, Y. (2022). Book language and its implications for children’s language, literacy, and development. *Current Directions in Psychological Science,**31*(4), 375–380. 10.1177/09637214221103264

[CR36] Neuman, S. B., Wong, K. M., Flynn, R., & Kaefer, T. (2019). Learning vocabulary from educational media: The role of pedagogical supports for low-income preschoolers. *Journal Of Educational Psychology,**111*(1), 32–44. 10.1037/edu0000278

[CR37] Neuman, S. B., Flynn, R., Wong, K., & Kaefer, T. (2020). Quick, incidental word learning in educational media: All contexts are not equal. *Educational Technology, Research and Development,**68*(6), 2913–2937. 10.1007/s11423-020-09815-z

[CR38] Newman, J., & Cox, C. (2020). Corpus Annotation. In M. Paquot & STh. Gries (Eds.), *A practical handbook of corpus linguistics* (pp. 25–48). Springer International Publishing. 10.1007/978-3-030-46216-1_2

[CR39] Ofcom. (2024). *Children and parents: Media use and attitudes report*. Ofcom. https://www.ofcom.org.uk/siteassets/resources/documents/research-and-data/media-literacy-research/children/children-media-use-and-attitudes-2024/childrens-media-literacy-report-2024.pdf?v=368229. Accessed 25 Sept 2024.

[CR40] Paetzold, G., & Specia, L. (2016). Collecting and Exploring Everyday Language for Predicting Psycholinguistic Properties of Words. In Y. Matsumoto & R. Prasad (Eds.), *Proceedings of COLING 2016, the 26th International Conference on Computational Linguistics: Technical Papers* (pp. 1669–1679). The COLING 2016 Organizing Committee. https://aclanthology.org/C16-1157/. Accessed 29 Jan 2025.

[CR41] R Core Team. (2023). *R: A language and environment for statistical computing. [Computer software]*. R Foundation for Statistical Computing. https://www.R-project.org/

[CR42] Rice, M. L., & Woodsmall, L. (1988). Lessons from television: Children’s word learning when viewing. *Child Development,**59*(2), 420–429. 10.2307/1130321 3359862 10.1111/j.1467-8624.1988.tb01477.x

[CR43] Rideout, V., & Robb, M. B. (2020). *The Common Sense Census: Media use by kids age zero to eight*. Common Sense Media. https://www.commonsensemedia.org/research/the-common-sense-census-media-use-by-kids-age-zero-to-eight-2020. Accessed 14 Oct 2022.

[CR44] Roettger, T. B. (2021). Preregistration in experimental linguistics: Applications, challenges, and limitations. *Linguistics,**59*(5), 1227–1249. 10.1515/ling-2019-0048

[CR45] Roseberry, S., Hirsh-Pasek, K., Parish-Morris, J., & Golinkoff, R. M. (2009). Live action: Can young children learn verbs from video? *Child Development,**80*(5), 1360–1375. 10.1111/j.1467-8624.2009.01338.x 19765005 10.1111/j.1467-8624.2009.01338.x PMC2759180

[CR46] Rowe, M. L. (2012). A longitudinal investigation of the role of quantity and quality of child-directed speech in vocabulary development. *Child Development,**83*(5), 1762–1774. 10.1111/j.1467-8624.2012.01805.x 22716950 10.1111/j.1467-8624.2012.01805.x PMC3440540

[CR47] Rowland, C. F., & Fletcher, S. L. (2006). The effect of sampling on estimates of lexical specificity and error rates. *Journal of Child Language,**33*(4), 859–877. 10.1017/S0305000906007537 17153864 10.1017/s0305000906007537

[CR48] Social Mobility Commission. (2024). *Distribution of parental education*. GOV.UK. https://social-mobility.data.gov.uk/drivers_of_social_mobility/conditions_of_childhood/distribution_of_parental_education/latest. Accessed 31 Jan 2025.

[CR49] Sönning, L., & Werner, V. (2021). The replication crisis, scientific revolutions, and linguistics. *Linguistics,**59*(5), 1179–1206. 10.1515/ling-2019-0045

[CR50] Temple Lang, D. (2024). *XML: Tools for Parsing and Generating XML Within R and S-Plus* (Version R package version 3.99-0.16.1) [Computer software]. https://CRAN.R-project.org/package=XML

[CR51] van Heuven, W. J. B., Mandera, P., Keuleers, E., & Brysbaert, M. (2014). Subtlex-UK: A new and improved word frequency database for British English. *Quarterly Journal of Experimental Psychology,**67*(6), 1176–1190. 10.1080/17470218.2013.850521 10.1080/17470218.2013.850521 24417251

